# Rural Residence, Nutrition Risk, and Falls In Community-Dwelling Older Adults

**DOI:** 10.1093/geroni/igaa057.764

**Published:** 2020-12-16

**Authors:** Nancy Gell, Caitlin Eckert, Jennifer Schollmeyer, Mariana Wingood, Emily Tarleton

**Affiliations:** 1 The University of Vermont, Burlington, Vermont, United States; 2 University of Vermont, Burlington, Vermont, United States; 3 Support and Services at Home, South Burlington, Vermont, United States; 4 University of Vermont, Waterbury, Vermont, United States; 5 Northern Vermont University, Johnson, Vermont, United States

## Abstract

Decreasing fall risk and maintaining independence is vital for community dwelling older adults. Nutritional status and rural residence may be independent predictors of falls. The aim of this study was to evaluate if nutritional status and rurality are positively associated with fall risk and predictive of future falls in community-dwelling older adults. We used data from a health risk assessment conducted by the Support and Services at Home organization serving Medicare beneficiaries in Vermont in 2017-2019 (N=3109; 79.6 years ±8.4, 75% female). Measures included the Fall Risk Questionnaire, Determine Nutrition Risk questionnaire, and fall history. Descriptive statistics from baseline measures and logistic regression analyses were used to identify predictors of a new fall with respect to rurality, fall risk, and nutritional status. At baseline, 67% of participants lived in rural communities, 37% had high nutrition risk, and 60% had elevated fall risk. Independently, rurality and high nutrition risk were significantly associated with fall risk (p<0.001) and high nutrition risk was associated with rurality (p<0.001). In the adjusted model, rural residence was not significantly associated with a fall within one year from baseline, whereas participants at high nutrition risk had a 50% higher odds of falling (p= 0.001). These findings suggest that falls may be associated with nutrition risk, but not living in a rural setting. Community-based initiatives should consider including nutrition screens as part of fall risk assessments. Further research is needed to understand the relationship between nutrition status and falls risk.

